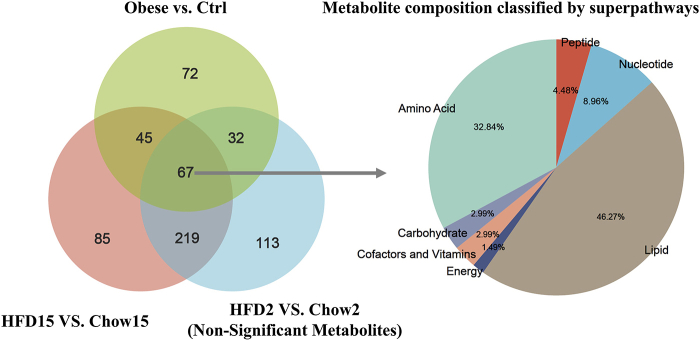# Tryptophan and polyamine metabolism dysregulation serves as an early marker of high-fat diet-induced glucose intolerance

**DOI:** 10.1016/j.jlr.2026.100980

**Published:** 2026-01-13

**Authors:** Jianfang Gao, Li Zhang, Shumin Zhan, Zhou Peng, Juan Du, Zhongxiao Zhang, Liling Xu, Shan Huang, Xingyun Wang, Xirong Guo

**Affiliations:** 1 Endocrinology Department, Tongren Hospital, Shanghai Jiao Tong University School of Medicine, Shanghai, China; 2 Hongqiao International Institute of Medicine, Tongren Hospital, Shanghai Jiao Tong University School of Medicine, Shanghai, China; 3 Department of Endocrinology, Children's Hospital, Zhejiang University School of Medicine, National Clinical Research Center for Child Health, Hangzhou, China

**Keywords:** dysglycemia, high-fat diet, metabolic perturbations, polyamine metabolism, tryptophan metabolism

## Abstract

A high-fat diet (HFD) induces metabolic dysfunction early, before the onset of the classic obese phenotype. However, understanding this early process remains limited, and potential diagnostic systems are still poorly investigated, particularly in childhood obesity. Continuous blood glucose monitoring was performed in mice to evaluate the early metabolic effects of HFD exposure. Metabolomic and transcriptomic analyses were conducted to characterize metabolic and transcriptional changes at various HFD feeding stages and investigate underlying mechanisms. Venn analysis was applied to identify metabolites specific to early HFD exposure. These metabolites were further compared with those detected in obese children to identify potential early warning biomarkers of obesity. Week 3 of HFD feeding was identified as a critical turning point in metabolic dysfunction in mice. Metabolomic profiling revealed that significant metabolic remodeling had occurred before glucose intolerance, particularly involving alterations in tryptophan metabolism, polyamine metabolism, and glycerophospholipid metabolism. Moreover, 54 HFD-specific metabolites were identified during this early stage. Further analysis identified serotonin, formiminoglutamate, inosine, and spermine as potential early warning biomarkers for HFD-induced obesity. Finally, transcriptomic profiling revealed early activation of interleukin-17A and type I interferon pathways, implicating immune involvement in metabolic perturbations. Early HFD exposure induces metabolic reprogramming before the onset of glucose intolerance. These_under_edi findings provide new insights into the mechanisms of diet-induced metabolic dysfunction and support the identification of potential biomarkers for early detection, particularly in childhood obesity. Early high-fat diet exposure induces metabolic reprogramming before glucose intolerance, characterized by alterations in tryptophan and polyamine metabolism and revealing candidate early biomarkers of obesity.

Childhood obesity_under_edi is a growing global health crisis with serious long-term consequences. It significantly increases the risk of metabolic dysfunction-associated steatotic liver disease, cardiovascular disease, and type 2 diabetes and imposes substantial psychosocial and economic burdens (1, 2, 3, 4). Notably, profound metabolic changes occur well before the appearance of classic obesity-related phenotypes (5). However, the limited understanding of these early events hampers insight into the spatiotemporal dynamics of obesity onset and the development of effective diagnostic systems. This knowledge gap contributes to the persistent rise in childhood obesity.

A high-fat diet (HFD) disrupts metabolic homeostasis by altering the expression and activity of key metabolic enzymes, transporters, and signaling molecules involved in glucose and lipid metabolism (6, 7). These disruptions worsen metabolic dysregulation and promote systemic imbalance. In particular, excessive dietary fat intake alters gut microbiota composition, leading to dysbiosis that impairs gut-living axis homeostasis. This disturbance affects hepatic metabolism through microbially derived or microbiota-dependent metabolites, such as secondary bile acids, short-chain fatty acids, and trimethylamine-*N*-oxide (8, 9, 10, 11). Progressive liver dysfunction results in hepatic insulin resistance and impaired insulin signaling, further exacerbating glucose intolerance. However, most research has focused on advanced stages of metabolic disease—such as established insulin resistance, obesity, type 2 diabetes, hepatic steatosis, and chronic inflammation (12, 13). Few studies have examined early glycemic dysregulation as a critical initiating event or investigated potential diagnostic systems from a metabolic perspective, particularly in the context of childhood obesity.

Therefore, the study aims to highlight the impact of early blood glucose changes and metabolic characteristics on the health of obese children with HFD. We measured fasting blood glucose levels, glucose tolerance test (GTT), and insulin tolerance test (ITT) at 2, 3, 4, 6, 8, and 15 weeks of HFD feeding mice. We revealed that week 3 was the key time point of HFD-induced metabolic dysfunction. To delineate the early metabolic changes before blood glucose abnormalities, we performed the metabolomic profiling of the 2-week, 3-week, and 15-week HFD feeding mice plasma samples by LC-MS analysis. We revealed the early characteristics of tryptophan metabolism, polyamine metabolism, and glycerophospholipid metabolic reprogramming. Finally, combined with plasma metabolomics in obese children, receiver operating characteristic (ROC) analysis showed the potential of serotonin, inosine, formiminoglutamate (FIGLU), and spermine as early warning biomarkers.

## Materials and Methods

### Animal experiments

All animal protocols are followed by the Animal Ethics Committee of Tongren Hospital, China (reference number: A2023-079-01, dated August 16, 2024). Mice were housed at 20–22°C under a 12 h light/dark cycle with ad libitum access to water and food. Four-week-old male C57BL/6J mice were obtained from GemPharmatech Laboratory and fed for 15 weeks on either a chow diet (15% kcal from fat; XTI01C, Xietong, China) or an HFD (60% kcal from fat, XTHF60, Xietong, China). During the experiments, both food intake and weight were recorded. Mice underwent fasting at 2, 3, 4, 6, 8, 10, and 15 weeks of HFD feeding, followed by intraperitoneal injections of glucose or insulin, after which blood glucose levels were measured at various time points. In addition, blood samples were collected at weeks 2, 3, and 15 for metabolomic profiling and peripheral blood mononuclear cell gene expression analysis.

### GTT and ITT

GTT was conducted at 2, 3, 4, 6, 8, 10, and 15 weeks of HFD treatment. Mice were fasted overnight (6–12 h) before receiving a glucose injection (2 g/kg body weight). For the ITT, mice were deprived of food for 4 h and then given an insulin injection at 0.75 IU per kilogram of body weight. Blood glucose levels were measured at 0, 30-, 60-, 90-, and 120 min postinjection for both tests. The glucose response was assessed by calculating the area under the curve (AUC) for blood glucose levels using the trapezoidal method.

### Body composition analysis

Briefly, at the 2, 3, 6, 8, 10, and 15 weeks of HFD feeding, body composition analysis was carried out in unanesthetized mice using an EchoMRI body composition analyzer (EchoMRI).

### Blood collection and peripheral blood mononuclear cell isolation

Blood was collected in a purple-top vacutainer, layered over Ficoll media, and centrifuged at 2,000 rpm for 20 min at 24°C. The upper layer of EDTA plasma was removed, divided into portions, and promptly frozen at 80°C. Subsequently, the mononuclear cell layer is carefully aspirated and transferred to a 50 ml centrifuge tube. The sample is diluted with PBS to a total volume of 50 ml and then centrifuged at 2000 rpm for 10 min. Subsequently, the pellet was resuspended and counted using a cell counter, and aliquots of peripheral blood mononuclear cells (PBMCs) were pelleted again and flash frozen. Then, we performed untargeted metabolomics analysis on plasma samples collected at weeks 2, 3, and 15 of HFD feeding. We performed transcriptomic analysis of PBMC gene expression only for the early stages of HFD feeding (weeks 2 and 3).

### Participants and blood sample collection

All participants were between 8 and 18 years old. Written informed consent was obtained from all child participants and their families before their involvement in the study, including permission for sample collection, usage, and data analysis. Ultimately, 30 patients diagnosed with obesity and 30 healthy individuals were included in the study. Participants' demographic and anthropometric information, including age, sex, weight, height, and BMI, was recorded. The diagnostic criteria for obesity followed the Chinese standard for overweight and obesity among school-age children and adolescents. Inclusion criteria were as follows: 1) age between 8 and 18 years and 2) diagnosis of obesity with the ability to provide informed consent. Exclusion criteria included 1) inability or unwillingness to provide informed consent; 2) a history or current diagnosis of type 1 diabetes mellitus; 3) the presence of any other chronic liver disease; and 4) failure to meet inclusion criteria.

Approximately 1 ml of blood was collected after an overnight fast of at least 8 h using EDTA-coated tubes. Then, the plasma samples were centrifuged at 2000 rpm for 15 min at low temperatures and analyzed using untargeted metabolomics. The study was approved by the Ethics Committee of the Children's Hospital, Zhejiang University School of Medicine (approval no.: 2021-IRB-301) and conformed to the Declaration of Helsinki.

### Untargeted UPLC-MS/MS analysis

Plasma samples were collected from chow-fed and HFD-fed mice at 2, 3, and 15 weeks (n = 10 per group) and analyzed by untargeted metabolomic profiling. Calibra Lab at DIAN Diagnostics in Hangzhou, Zhejiang, China, conducted an untargeted metabolomic study using the CalOmics metabolomics platform. The extraction of samples was done using methanol in a one-fourth proportion. The mixtures were agitated for 3 min and then separated by centrifuging at 4,000 *g* for 10 min at 20°C. Following Calibra Technology Co, Ltd (Hangzhou, China) protocols, four 100 μl supernatant aliquots were placed in sample plates, dried under nitrogen, and redissolved for UPLC-MS/MS system injection. The four UPLC-MS/MS methods utilize the ACQUITY 2D UPLC from Waters (Milford, MA) and the Q Exactive hybrid Quadrupole-Orbitrap mass spectrometer from Thermo Fisher Scientific (San Jose, CA). Statistical analysis was performed in R software (version 3.4.1) and the mixOmics package (version 6.10.9). Statistical methods, parametric (such as Student's *t*-test and ANOVA) and nonparametric (like Wilcox's rank test and Kruskal-Wallis test), identified significant metabolite changes between HFD and chow feeding groups. Metabolites were identified as differential if their *P* value was below 0.05. Metabolic enrichment and pathway analysis, utilizing Kyoto Encyclopedia of Genes and Genomes database searches, identified and integrated the differing metabolites between the two groups into their respective biochemical pathways.

### Immunoblotting

Adipose tissue was extracted with RIPA buffer with 50X protease and phosphatase inhibitor cocktail (Beyotime, China). Protein was quantified using a BCA Protein Assay Kit. A total of 30 mg protein was loaded into a 12% SDS-PAGE gel and transferred onto a polyvinylidene difluoride membrane (pore size: 0.22 mm; Millipore). Next, they were overnight applied with primary antibodies: AADAT (Ablybio, B19480, 1:1,000-1:5,000 dilution, rabbit polyclonal), ODC1 (Proteintech, 28728-1-AP, 1:500-1:1,000 dilution, rabbit polyclonal), SPMS (Abways, DY1503, 1:1,000-1:2,000 dilution, rabbit monoclonal), SPDS (Cohesionbio, CQA1738, 1:500-1:2,000 dilution, rabbit polyclonal), TPH2 (Proteintech, 29283-1-AP, 1:500-1:1,000 dilution, rabbit polyclonal), and β-actin (Proteintech, 20536-1-AP, 1:5,000 dilution, rabbit polyclonal). Subsequently, membranes were washed with Tris-buffered saline with Tween-20 buffer and incubated with secondary antibodies. After additional washing with Tris-buffered saline with Tween-20, the membranes were developed using ECL (Tanon, China) and quantified with ImageJ software (National Institutes of Health, Bethesda, MD).

### RNA extraction and RNA sequencing from PBMCs

PBMCs were thawed on ice, followed by total RNA isolation using TRIzol reagent (Takara, 9109, Japan) and subsequent qualification and purification with a NanoDrop ND-1000 spectrophotometer (NanoDrop Technologies). RNA integrity number greater than 7.0 using the Bioanalyzer 2100 (Agilent Technologies, CA). Denaturing agarose gel electrophoresis was performed to confirm RNA integrity, utilizing 1 μg of RNA per sample for preparation. Sequencing libraries were prepared following the manufacturer's protocol using the NEBNext® Ultra^TM^ RNA Library Prep Kit for Illumina® (NEB), incorporating index codes for sample-specific sequence allocation. mRNA was isolated from total RNA using poly-T oligo-conjugated magnetic beads. The fragmentation process utilized divalent cations and was conducted at high temperatures in the NEBNext First Strand Synthesis Reaction Buffer (5X). First-strand complementary DNA (cDNA) synthesis was performed using a random hexamer primer and M-MuLV Reverse Transcriptase (RNase H-), followed by second-strand synthesis with DNA Polymerase I and RNase H. Exonuclease and polymerase activities were employed to convert residual overhangs into blunt ends. The 3′ ends of DNA fragments were adenylated, followed by ligation of the NEBNext Adaptor with a hairpin loop structure to prepare for hybridization. The library fragments were purified using the AMPure XP system (Beckman Coulter, Beverly, CA) to select cDNA fragments, ideally ranging from 250 to 300 bp in length. The AMPure XP system (Beckman Coulter, Beverly) was used to purify cDNA library fragments, selecting those preferentially 250–300 bp in length. The size-selected, adaptor-ligated cDNA was treated with 3 μl of USER Enzyme (NEB) at 37°C for 15 min, followed by heating at 95°C for 5 min before PCR. PCR was conducted using Phusion High-Fidelity DNA polymerase, Universal PCR primers, and Index (X) Primer. The AMPure XP system was used to purify the PCR products, and the Agilent Bioanalyzer 2100 system assessed the library quality.

Differential expression analysis for two groups/conditions, each having two biological replicates, was executed using the R package (1.16.1). DESeq2 employs statistical techniques to detect differential expression in digital gene expression data using a negative binomial distribution model. Significant differential expression was determined using a *P* value threshold of 0.05 and an absolute fold change of 2.

### Statistical analysis

Statistical analyses were performed using GraphPad Prism software, employing unpaired two-tailed Student's *t*-test for comparisons between two groups and two-way ANOVA for comparisons involving more than two groups, followed by Tukey's multiple comparisons test when necessary. Pearson's correlation analysis was used to investigate the association between BMI and metabolites. An ROC curve was constructed using the predicted probability of an obesity diagnosis as a surrogate marker. The area under the ROC curve, along with its 95% confidence intervals, was used as an accuracy index to assess the diagnostic performance of obesity. Data are expressed as mean ± SEM or SD, with statistical significance defined as *P* < 0.05.

## Results

### Short-term HFD-induced metabolic dysfunction highlights week 3 as a pivotal time point

**Fig. 1:**
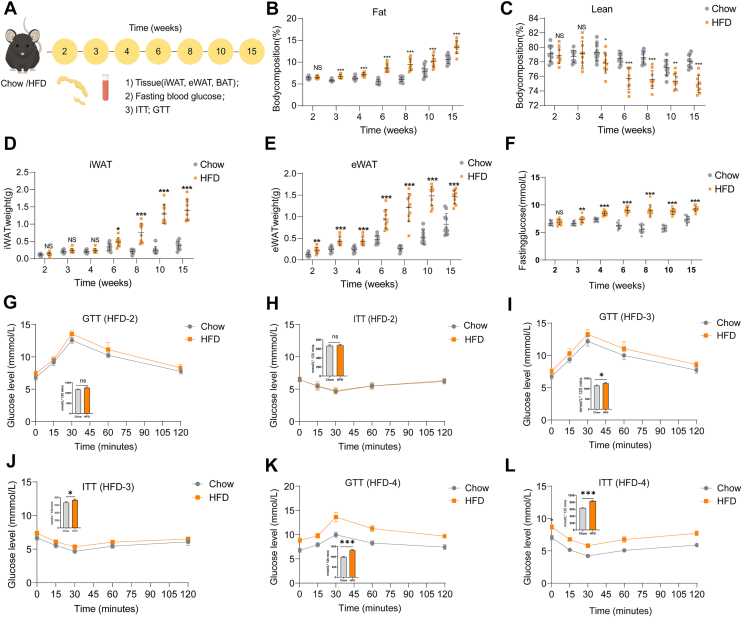
**Week 3 of HFD feeding marks a turning point in glucose dysregulation.** A: Schematic illustration of the experimental timeline for HFD feeding and metabolic assessments. Four-week-old male C57BL/6J mice were fed a chow (n = 10) or HFD (n = 10) for 15 weeks. B: Body composition showing fat mass. C: Body composition showing lean mass. D: IWAT mass (g). E: eWAT mass (g). F: Fasting blood glucose in chow and HFD mice. G: GTT in chow and HFD mice at 2 weeks. The inset shows AUC. H: ITT at 2 weeks. The inset shows AUC. I: GTT in chow and HFD mice at 3 weeks. The inset shows AUC. J: ITT at 3 weeks. The inset shows AUC. K: GTT in chow and HFD mice at 4 weeks. The inset shows AUC. L: ITT at 4 weeks. The inset shows AUC. Each symbol represents an individual mouse. Data are expressed as mean ± SEM or SD, ∗*P* < 0.05, ∗∗*P* < 0.01, ∗∗∗*P* < 0.001 versus chow. iWAT, inguinal adipose tissue; NS, not significant.

**Supplemental Figure S1:**
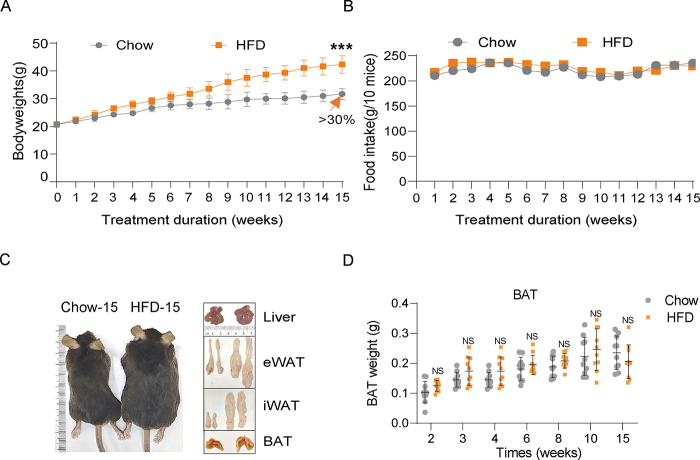


To reveal the early term impacts of HFD on systemic metabolic homeostasis, 4-week-old mice were fed with HFD for 15 weeks. Body weight, adipose tissue weights, fasting blood glucose levels, ITT, and GTT were measured at 2, 3, 4, 6, 8, and 15 weeks of HFD (Fig. 1A). Indeed, after a 15-week feeding, the weight of high-fat-fed mice increased by 30% (Supplemental Fig. S1A). As expected, caloric intake was greater in the HFD-fed mice than in the chow group (Supplemental Fig. S1B). Body composition analysis revealed a significant increase in fat mass after 3 weeks of HFD feeding, whereas lean mass began to decline from week 4 (Fig. 1B and C). Inguinal white adipose tissue weight markedly increased in HFD-fed mice starting from week 4 (Fig. 1D). Moreover, epididymal adipose tissue (eWAT) weight was already elevated after 2 weeks of HFD feeding (Fig. 1E). Notably, the weight of interscapular brown adipose tissue (BAT) did not change significantly throughout (Supplemental Fig. S1D).

**Supplemental Figure S2:**
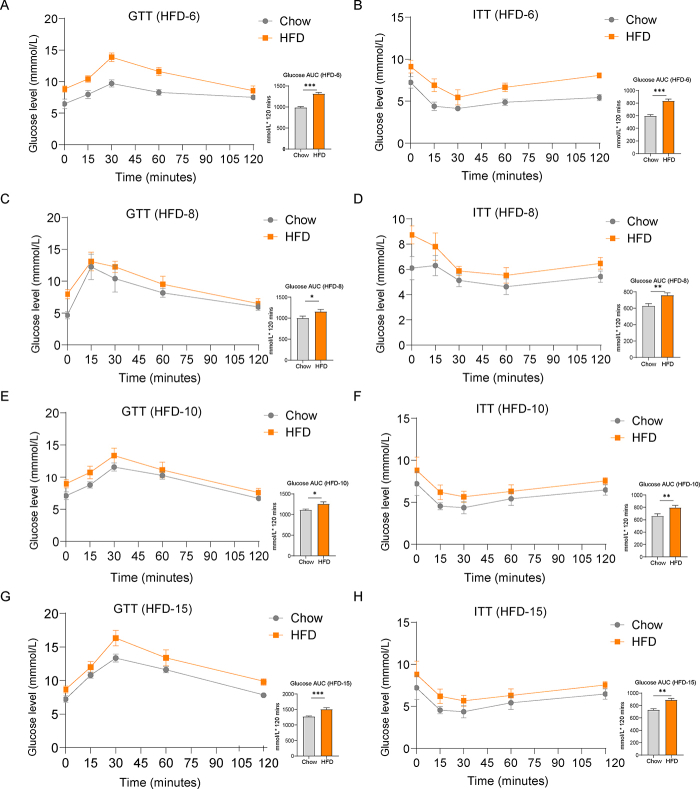


Glucose metabolic dysfunction is one of the earliest events in HFD-induced metabolic impairment. Here, we aimed to understand the critical early time points underlying these initial metabolic disturbances. Blood glucose measurements showed that fasting glucose levels remained comparable to those of the chow-fed group during the first 2 weeks of HFD feeding (Fig. 1G and H). Starting from week 3, however, HFD-fed mice exhibited impairments in both insulin and glucose tolerance compared with chow-fed controls, indicating that 3 weeks of HFD feeding marks a pivotal turning point for the onset of early metabolic dysfunction (Fig. 1I–L, Supplemental Fig. S2). Consistently, our study confirmed that 3 weeks of HFD feeding marks a key time point for the development of early metabolic dysfunction.

### HFD disturbed the plasma metabolic profile preceding glucose dysregulation

**Fig. 2:**
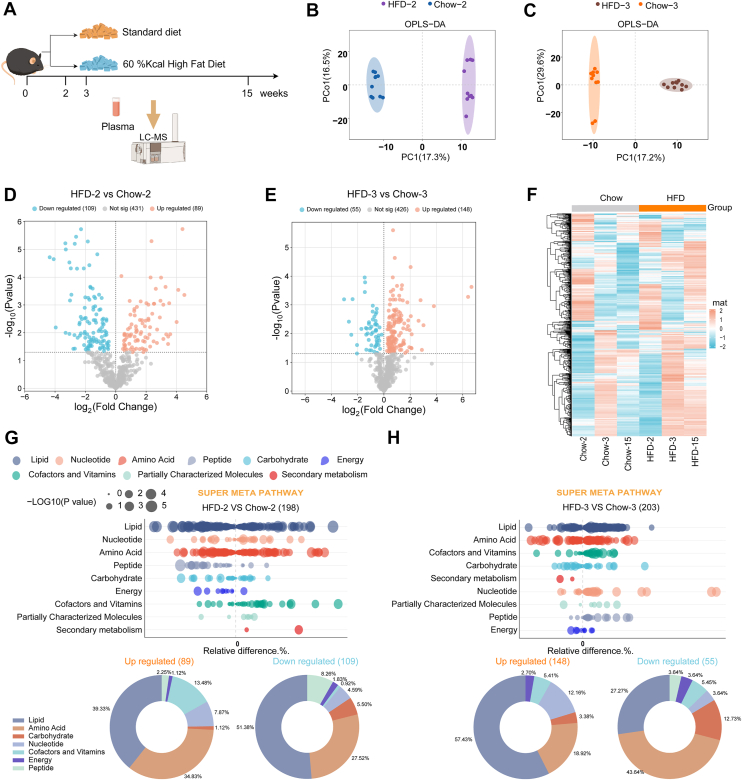
**HFD-fed mice exhibit an altered metabolome preceding glucose dysregulation.** A: Schematic of the experimental design. Mice were fed with chow or HFD, and plasma samples were collected at weeks 2, 3, and 15 for metabolomic analysis via LC-MS. B and C: The OPLS-DA plots of plasma metabolites in C57BL/6 mice fed with a chow or HFD for 2 weeks (B) and 3 weeks (C). Each data point represents an individual mouse. D and E: Volcano plots of differential metabolites that were upregulated (yellow) or downregulated (blue) in plasma samples (HFD group vs. chow group) at week 2 (D) and week 3 (E). F: Heatmap for significantly changed metabolites between the HFD and chow group. G and H: Biological classifications of altered metabolites at weeks 2 (G) and 3 (H) were categorized by superclasses, such as lipids, amino acids, carbohydrates, and cofactors. A bubble plot shows the relative differences in the detected metabolites. The following donut charts illustrate the class distribution of upregulated and downregulated metabolites (left donuts, upregulated; right donuts, downregulated). OPLS-DA, orthogonal partial least squares-discriminate.

**Supplemental Figure S3:**
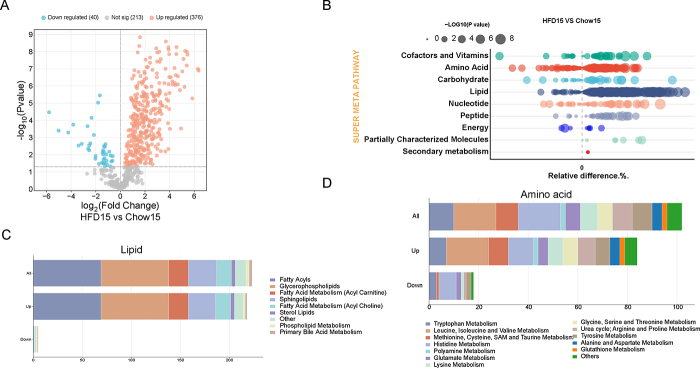


To uncover the early impacts of HFD on systemic metabolic homeostasis, we conducted untargeted metabolomics on serum from the chow-fed and HFD-fed for 2 weeks, 3 weeks, and 15 weeks (n = 10), and a mass-based comprehensive computational framework, 706 features were detected (Fig. 2A). The partial least squares-discriminate analysis plot of differential metabolites showed significant variations between the metabolome of mice fed chow and HFD for 2 weeks and 3 weeks (Fig. 2B and C). We then statistically evaluated the ion feature matrix. Potential biomarkers were identified based on *P* < 0.05. At week 2, 89 metabolites were upregulated and 109 were downregulated (Fig. 2D). In contrast, at week 3, 203 metabolites were significantly altered, including 148 upregulated and 55 downregulated (Fig. 2E). Furthermore, after 15 weeks of HFD feeding, most differential metabolites were upregulated (Supplemental Fig. S3A), revealing that short-term HFD exposure (HFD2) disturbed the plasma metabolic profile prior to the onset of glucose dysregulation (HFD3).

We speculated that significant metabolic reprogramming might be involved in the in vivo early metabolic damage. Heatmap analysis revealed extensive alterations in metabolite abundance in the HFD group (Fig. 2F), which coincides with the previous result showing that consumption of HFD is a significant factor in developing these metabolic disturbances. We further annotated the HFD-induced changes in the biological classification of the altered metabolites, revealing that amino acids and lipids were most affected at every time point (Fig. 2G and H, Supplemental Fig. S3B–D).

### Early differential metabolic patterns classified by lipids

**Fig. 3:**
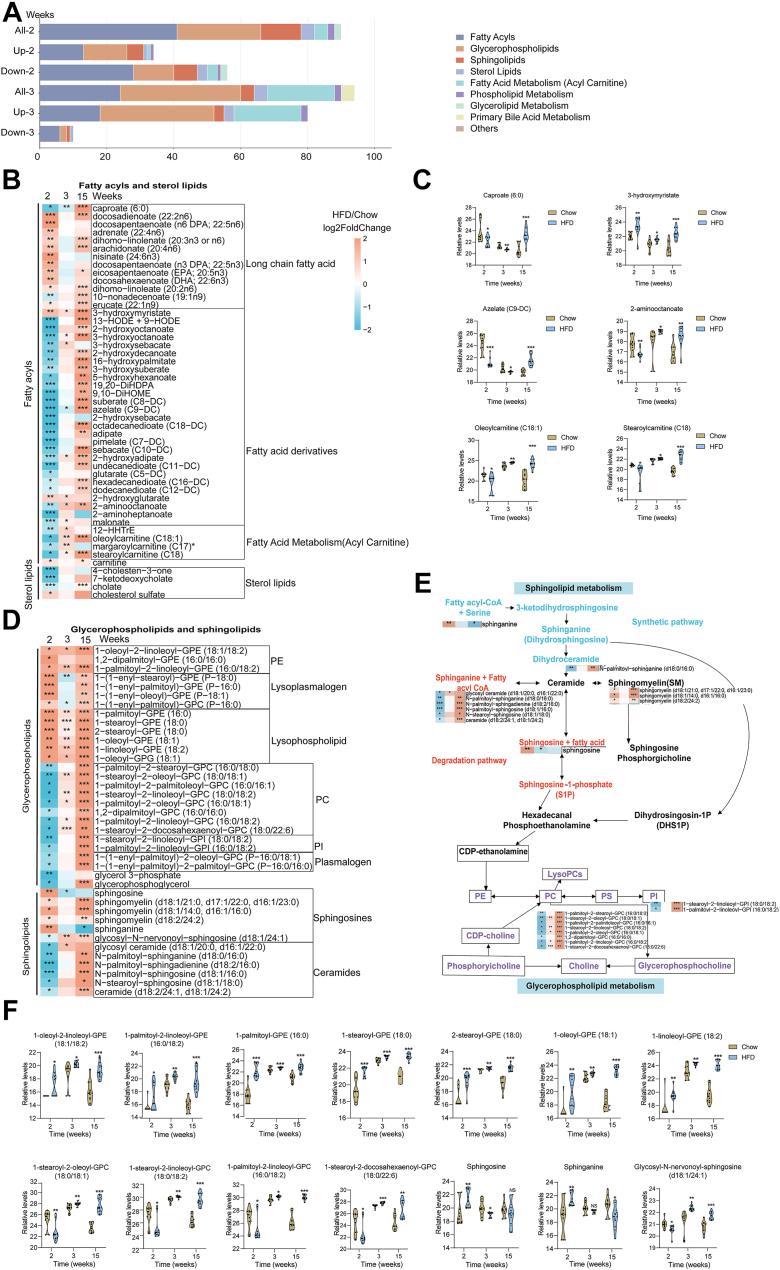
**Early lipid metabolic features preceding glucose dysregulation under HFD.** A: Classification of altered lipid subclasses at weeks 2 and 3 based on relative abundance and direction of change. B: Heatmap showing the temporal changes of early differentially altered fatty acyls and sterol lipids in plasma. Long-chain fatty acids, fatty acid derivatives, acylcarnitines, and sterol lipids were dynamically altered in response to HFD. C: Relative levels of early altered metabolites related to fatty acyls and sterol lipids in mice at different stages. D: Heatmap showing the temporal changes of early differentially altered glycerophospholipids and sphingolipids. E: Graphical representation of the glycerophospholipid and sphingolipid metabolic pathways. Yellow-filled and blue squares indicate metabolites increased and decreased in HFD, respectively. F: Relative levels of early altered metabolites related to glycerophospholipids and sphingolipid metabolism in mice at different stages. Data are expressed as mean ± SD. ∗*P* < 0.05, ∗∗*P* < 0.01, ∗∗∗*P* < 0.001 versus chow, NS, not significant.

To clarify early metabolic reprogramming, we focused on the 2-week HFD group in subsequent analyses. In our study, we identified plasma lipid signatures through 335 annotated lipids. Among these lipid species, we observed that the fatty acyls, glycerophospholipids, sphingolipids, fatty acid metabolism (acylcarnitine), and sterol lipids came out on top 5 in the list of altered metabolism pathways under HFD (Fig. 3A). Heatmap analysis revealed early lipid metabolic disturbances during HFD feeding, characterized by upregulation of long-chain fatty acids and downregulation of fatty acid derivatives, acylcarnitines, and sterol lipids. Notably, 3-hydroxymyristate was markedly increased, whereas caproate (C6:0), azelate (C9-DC), 2-aminooctanoate, oleoylcarnitine (C18:1), and stearoylcarnitine (C18:0) were decreased, indicating reduced mitochondrial fatty acid transport and β-oxidation in HFD-feeding mice (Fig. 3B and C). Moreover, subclasses of glycerophospholipids and sphingolipids, including phosphatidylcholines, phosphatidylinositols, plasmalogens, and ceramides, were consistently downregulated under HFD conditions. In contrast, phosphatidylethanolamines, lysophospholipids, and sphingosines were upregulated. Notably, nearly all early differential metabolites related to glycerophospholipids and sphingolipids were upregulated after 15 weeks of HFD feeding (Fig. 3D). This is consistent with the development of hyperglycemia and systemic inflammation commonly observed during prolonged HFD feeding. Metabolic pathway mapping revealed that 2-week HFD disrupted key steps in sphingolipid and glycerophospholipid metabolism, including ceramide and sphingomyelin synthesis, as well as CDP-choline and CDP-ethanolamine pathways, indicating reduced phospholipid remodeling capacity. Longitudinal tracking of representative metabolites confirmed that these lipid perturbations persisted or worsened over time (Fig. 3E and F).

### Differential metabolic patterns during HFD classified by amino acids

**Fig. 4:**
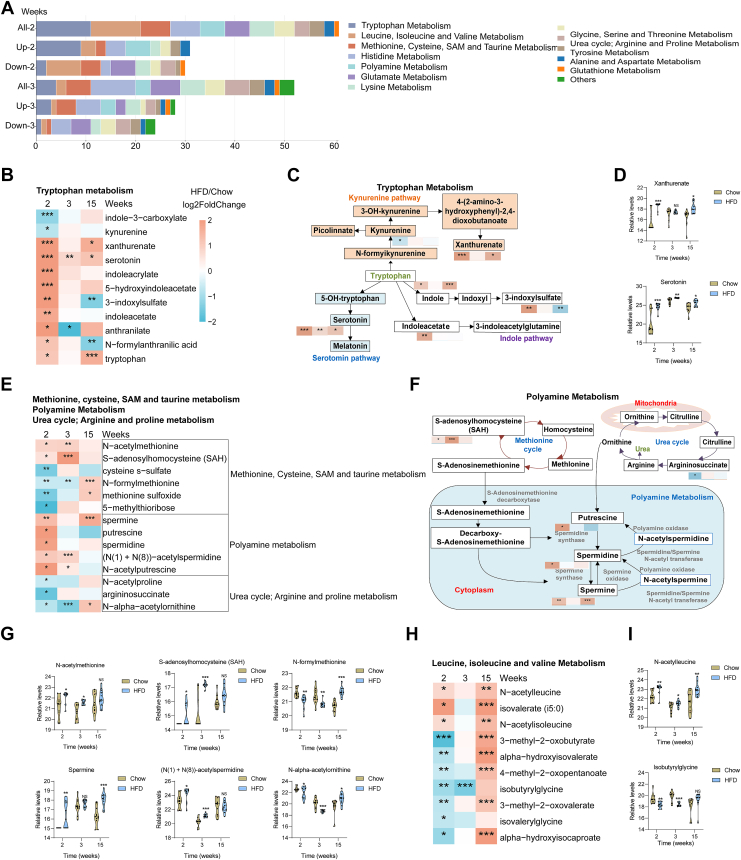
**Early amino acid metabolic features preceding glucose dysregulation under HFD.** A: Classification of altered amino acid subclasses at weeks 2 and 3 of HFD feeding based on relative abundance and direction of change. B: Heatmap showing the temporal changes of early differentially altered metabolites related to tryptophan metabolism. C: Graphical representation of the tryptophan metabolism pathway. Red-filled and blue squares indicate metabolites increased and decreased in HFD, respectively. D: Relative levels of early altered metabolites related to tryptophan metabolism at different stages. E: Heatmap showing the temporal changes of early differentially altered metabolites related to methionine, cysteine, SAM, taurine metabolism, polyamine metabolism, the urea cycle, and arginine and proline metabolism. F: Graphical representation of the interconnected polyamine, urea cycle, and methionine metabolic network, showing differential regulation under HFD. G: Relative levels of representative metabolites. H and I: Heatmap (H) and relative levels of representative metabolites (I) of BCAA metabolism at different stages. Data are expressed as mean ± SD. ∗*P* < 0.05, ∗∗*P* < 0.01, ∗∗∗*P* < 0.001 versus chow. NS, not significant; SAM, S-adenosylmethionine.

**Supplemental Figure S4:**
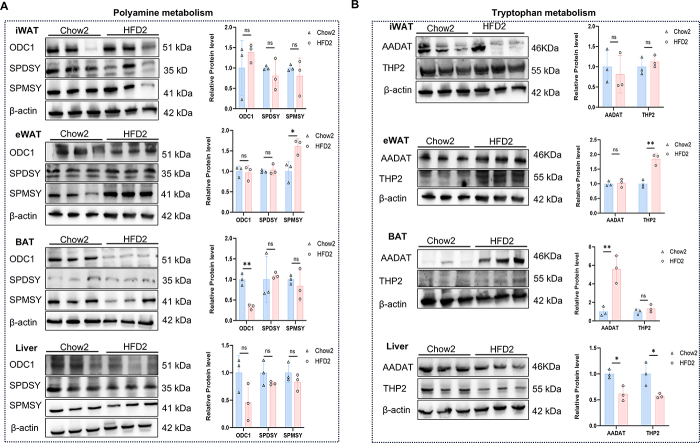


HFD feeding induced pronounced remodeling of amino acid metabolism. Early responses were observed in tryptophan metabolism, polyamine metabolism, methionine/cysteine/S-adenosylmethionine cycle, and branched-chain amino acid (BCAA) metabolism (Fig. 4A). Specifically, 11 tryptophan-derived metabolites showed significant alterations as early as week 2 of HFD feeding. Of these metabolites, 2 were significantly decreased by HFD feeding, such as kynurenine and indole-3-carboxylate. In contrast, 9 metabolites exhibited concordant changes, with increases observed in xanthurenate, serotonin, and 5-hydroxyindoleacetate (Fig. 4B). Metabolic pathway mapping revealed global activation of the kynurenine, serotonin, and indole branches of tryptophan metabolism, indicating enhanced immunomodulatory metabolic fluxes. Consistent with this, circulating levels of serotonin and indoleacetate exhibited an early increase in serotonin, followed by a sustained accumulation of indole-derived metabolites (Fig. 4C and D). In parallel, metabolites within the polyamine pathway, such as spermidine, putrescine, and *N*-acetylated derivatives, were significantly upregulated as early as week 2 (Fig. 4E and F), indicating a preferential shift of ornithine toward polyamine synthesis over urea production. This is confirmed through quantitative profiling of polyamine intermediates (Fig. 4G). Next, we examined the expression of polyamine metabolism-related enzymes (ODC1, SPDSY, and SPMSY) and tryptophan metabolism-related enzymes (THP2 and AADAT) in adipose tissues and liver after early HFD feeding (Supplemental Fig. S4). The results revealed that metabolic reprogramming of both polyamine and tryptophan pathways primarily occurs in adipose tissues—particularly in eWAT and BAT.

Additionally, in contrast to the early tryptophan and polyamine mobilization, BCAA catabolism was impaired after 2 weeks of HFD feeding. Several catabolites derived from leucine, isoleucine, and valine, including isobutyrylglycine, isovalerylglycine, 3-methyl-2-oxobutyrate, and 4-methyl-2-oxopentanoate, were significantly downregulated from week 2 onward (Fig. 4H). In contrast, *N*-acetylleucine and isovalerate (i5:0) showed a consistent elevation trend across all time points (Fig. 4I). HFD feeding induces coordinated metabolic reprogramming of amino acid pathways, characterized by early tryptophan and polyamine metabolism activation.

### Metabolites uniquely altered by early HFD

**Fig. 5:**
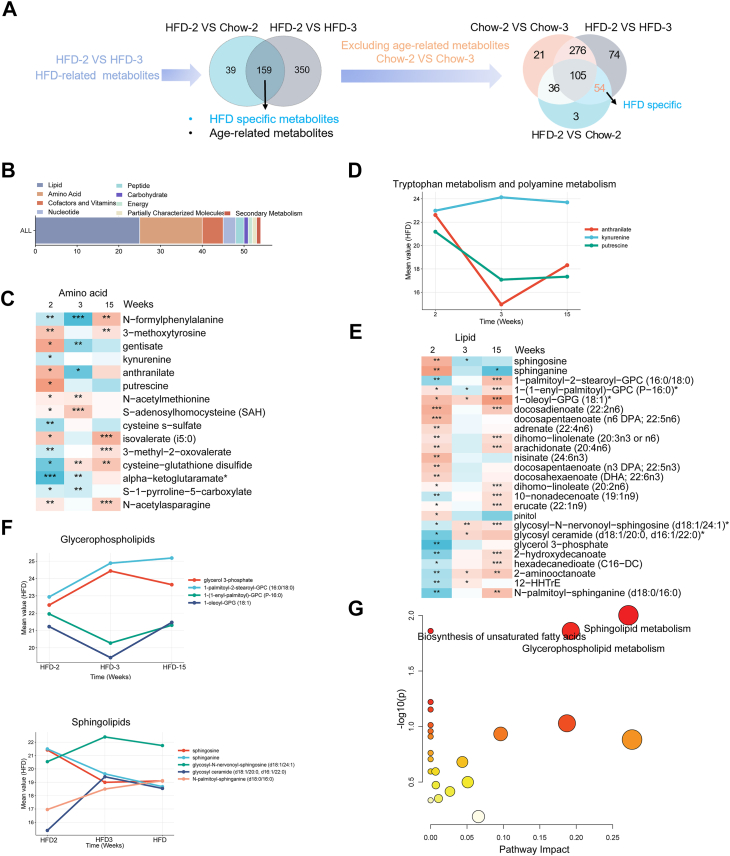
**HFD-specific metabolites.** A: Venn diagram displaying HFD unique differential metabolites across comparisons: HFD2 versus Chow2, HFD3 versus Chow3, and HFD3 versus HFD2. B: Distribution of HFD-specific differential metabolites classified by super pathways. C: Heatmap of HFD-specific amino acid-related metabolites altered at different stages. D: Relative levels of representative metabolites in the HFD group at 2, 3, and 15 weeks of feeding. E: Heatmap of HFD-specific lipid-related metabolites altered at different stages. F: Relative levels of representative metabolites in the HFD group at 2, 3, and 15 weeks of feeding. G: Pathway impact analysis of HFD-specific metabolites, highlighting key metabolic pathways with significant alterations.

To identify early metabolic alterations induced by HFD, we conducted differential metabolomic comparisons between HFD3 versus HFD2 and HFD2 versus Chow2, identifying 159 overlapping metabolites. After excluding 105 age-related changes (as determined by Chow3 vs. Chow2), a total of 54 HFD-specific metabolites were defined (Fig. 5A, Table 1), with the majority belonging to lipid and amino acid classes (Fig. 5B). Among amino acid-related changes (Fig. 5C), early alterations in tryptophan and polyamine-derived metabolites, such as kynurenine, anthranilate, and putrescine, indicate perturbations in the kynurenine and polyamine pathways under HFD conditions. In parallel, BCAA-related intermediates, including 3-methyl-2-oxovalerate, exhibited suppressed levels. On the other hand, lipid metabolism exhibited prominent remodeling (Fig. 5D). Specifically, early increases were observed in sphingolipids (sphingosine, sphinganine) and glycerophospholipids, such as 1-(1-enyl-palmitoyl)-GPC(P-16:0), 1-oleoyI-GPG (18:1), along with progressive accumulation of polyunsaturated fatty acids (docosahexaenoate, arachidonate, and docosapentaenoate) (Fig. 5E and F). These alterations suggest active membrane lipid remodeling and early lipid signaling dysregulation.

**Table 1:** HFD-specific metabolites

HMDB	Metabolites	Super meta pathway	HFD-2/chow-2^a^			HFD-3/chow-3^a^		
			VIP	FC	*P*	VIP	FC	*P*
HMDB0006028	*N*-acetylasparagine	Amino acid	0.5,518,624	2.04,560,233	0.0029	0.206,219,556	0.67,115,287	0.15545
HMDB0001552	Alpha-ketoglutaramate	Amino acid	1.554,170,656	0.03,508,891	0.0344	0.666,972,496	0.5,899,136	0.0077
HMDB0001301	*S*-1-pyrroline-5-carboxylate	Amino acid	0.198,588,213	0.65,357,284	0.0467	0.36,436,894	0.56,246,529	0.01725
HMDB0000656	Cysteine-glutathione disulfide	Amino acid	0.444,477,227	0.28,091,129	0.0257	1.199,366,156	2.28,789,548	6.2E-05
HMDB0000491	3-Methyl-2-oxovalerate	Amino acid	0.633,740,464	0.49,114,463	0.0225	0.022,314,358	1.14,087,342	0.39002
HMDB0000718	Isovalerate (i5:0)	Amino acid	2.111,404,873	9.68,463,662	0.0271	0.246,238,031	0.68,094,844	0.03329
HMDB0000731	Cysteine *s*-sulfate	Amino acid	0.603,332,573	0.26,135,571	0.0025	0.257,608,263	0.79,130,057	0.65577
HMDB0011745	*N*-acetylmethionine	Amino acid	0.426,020,782	1.7,135,339	0.0183	0.461,958,074	1.84,837,008	0.00053
HMDB0000939	*S*-adenosylhomocysteine	Amino acid	0.031,036,807	2.09,954,384	0.039	0.119,248,099	3.22,196,196	4.3E-05
HMDB0001414	Putrescine	Amino acid	1.655,068,737	29.1,314,752	0.0146	0.137,770,391	0.45,930,917	0.04694
HMDB0001123	Anthranilate	Amino acid	0.330,829,891	1.30,740,202	0.608	0.238,495,975	0.08,974,597	0.00682
HMDB0000684	Kynurenine	Amino acid	0.706,109,009	0.54,525,657	0.0126	0.31,787,805	1.09,096,598	0.6839
HMDB0001434	3-Methoxytyrosine	Amino acid	0.320,112,358	2.95,764,608	0.0037	0.078,404,003	0.81,660,657	0.42355
HMDB0000152	Gentisate	Amino acid	0.476,204,546	49.8,167,283	0.018	0.090,996,321	0.13,330,912	0.00707
HMDB0240317	*N*-formylphenylalanine	Amino acid	0.309,008,954	0.50,690,222	0.0032	0.516,078,402	0.11,316,658	0.0039
HMDB0000174	Fucose	Carbohydrate	0.866,134,272	0.58,206,412	0.0515	1.078,148,913	1.2,362,135	0.06856
HMDB0240294	2-*O*-methylascorbic acid	Cofactors and Vitamins	1.176,645,383	2.11,119,633	0.0103	0.416,380,819	0.59,819,398	0.24202
HMDB0004194	N1-Methyl-4-pyridone-3-carboxamide	Cofactors and vitamins	1.736,791,699	2.0013758	0.0067	0.315,314,309	0.96,899,234	0.90279
HMDB0000210	Pantothenate	Cofactors and vitamins	1.725,405,334	2.14,443,738	0.0113	0.227,491,543	0.98,356,568	0.89993
HMDB0000235	Thiamin (vitamin B1)	Cofactors and vitamins	0.592,406,751	3.43,922,273	0.0021	0.174,970,506	0.64,549,131	0.21315
HMDB0000017	Pyridoxate	Cofactors and vitamins	1.058,211,391	1.97,967,354	0.0156	0.052,424,892	0.95,011,955	0.87492
HMDB0001429	Phosphate	Energy	3.002,413,813	1.68,757,552	0.0278	4.078,809,771	0.37,724,006	0.01843
HMDB0011760	*N*-palmitoyl-sphinganine (d18:0/16:0)	Lipid	0.168,650,374	0.2,207,007	0.0063	0.045,916,211	0.80,268,993	0.48055
HMDB0012535	12-HHTrE	Lipid	0.334,567,535	0.48,999,707	0.0198	0.460,362,464	1.68,084,851	0.02988
HMDB0000991	2-Aminooctanoate	Lipid	0.095,313,475	0.36,911,159	0.0138	0.17,896,945	1.7,915,786	0.00149
HMDB0000672	Hexadecanedioate (C16-DC)	Lipid	0.200,097,905	0.59,085,536	0.0189	0.207,781,301	1.37,678,478	0.15188
HMDB0094656	2-Hydroxydecanoate	Lipid	0.316,588,602	0.18,247,852	0.0047	0.17,766,245	1.32,733,618	0.07861
HMDB0000126	Glycerol 3-phosphate	Lipid	1.386,121,251	0.12,199,487	0.0285	1.284,634,088	0.3,657,656	0.03344
HMDB0004973	Glycosyl ceramide (d18:1/20:0, d16:1/22:0)	Lipid	0.130,465,171	0.09,556,928	0.0378	0.19,181,397	1.88,088,915	0.00157
HMDB0004975	Glycosyl-*N*-nervonoyl-sphingosine (d18:1/24:1)	Lipid	0.187,974,073	0.66,659,588	0.0283	0.702,125,104	2.18,383,112	4.6E-05
HMDB0034219	Pinitol	Lipid	0.147,431,637	1.89,867,898	0.0102	0.092,043,137	0.50,739,582	0.04293
HMDB0013622	10-Nonadecenoate (19:1n9)	Lipid	0.771,768,869	0.62,560,106	0.009	0.298,661,942	1.12,805,881	0.47658
HMDB0002068	Erucate (22:1n9)	Lipid	0.72,005,652	1.4,529,088	0.0503	0.018,809,608	0.9,060,976	0.65386
HMDB0002925	Dihomo-linolenate (20:3n3 or n6)	Lipid	3.742,152,857	2.05,123,759	0.0032	2.461,620,379	0.62,611,637	0.08618
HMDB0240602	1-Oleoyl-GPG (18:1)	Lipid	0.537,481,246	5.36,021,534	0.0278	0.157,931,292	1.69,465,996	0.03397
HMDB0010407	1-(1-Enyl-palmitoyl)-GPC (P-16:0)	Lipid	0.430,678,863	2.02,303,755	0.0233	0.213,689,746	0.66,837,546	0.01059
HMDB0007970	1-Palmitoyl-2-stearoyl-GPC (16:0/18:0)	Lipid	0.942,752,438	0.33,355,715	0.0011	0.145,127,745	1.0766838	0.59273
HMDB0002226	Adrenate (22:4n6)	Lipid	3.12,079,641	2.50,039,406	0.0011	0.349,316,743	0.99,320,321	0.9581
HMDB0001043	Arachidonate (20:4n6)	Lipid	11.31,709,072	2.44,063,046	0.0025	6.469,213,223	0.66,062,293	0.07803
HMDB0005060	Dihomolinoleate (20:2n6)	Lipid	1.976,582,786	1.56,343,012	0.0089	0.147,973,413	1.00695045	0.96532
HMDB0061714	Docosadienoate (22:2n6)	Lipid	2.113,768,814	11.8,950,687	0.0011	0.086,152,757	1.09,366,303	0.55832
HMDB0002183	DHA (22:6n3)	Lipid	9.729,315,987	2.42,588,776	0.0114	5.436,185,012	0.60,972,613	0.08353
HMDB0006528	docosapentaenoate (n3; 22:5n3)	Lipid	3.533,088,828	2.93,477,709	0.0061	1.048,027,557	0.73,096,166	0.12976
HMDB0001976	docosapentaenoate (n6; 22:5n6)	Lipid	4.721,698,275	3.10,911,459	0.0015	0.746,273,502	0.88,845,299	0.5248
HMDB0002007	Nisinate (24:6n3)	Lipid	1.436,701,719	3.29,757,737	0.0016	0.454,569,957	0.70,648,547	0.18923
HMDB0000269	Sphinganine	Lipid	0.448,302,045	3.02,029,132	0.0571	0.266,115,398	0.44,671,746	0.0493
HMDB0000252	Sphingosine	Lipid	0.507,550,201	3.63,185,551	0.0537	0.30,595,137	0.28,388,671	0.0378
HMDB0061711	Methylphosphate	Nucleotide	0.133,978,967	1.3,493,641	0.0002	0.140,195,006	0.6,695,814	0.0966
HMDB0000045	AMP	Nucleotide	0.113,862,994	0.38,518,902	0.012	0.031,848,419	0.69,627,328	0.61199
HMDB0002031	3-Ureidoisobutyrate	Nucleotide	0.260,312,561	0.01,360,541	0.0664	0.032,100,388	1.46,768,853	0.14351
HMDB0000892	Pentose acid	Partially characterized molecules	0.285,223,528	1.89,666,578	0.007	0.276,750,214	0.34,373,797	0.00384
HMDB0011171	Gamma-glutamylleucine	Peptide	0.522,749,405	0.28,839,966	0.0063	0.059,804,732	1.0377274	0.79994
HMDB0034367	Gamma-glutamylmethionine	Peptide	0.518,903,413	0.03,911,877	0.0699	0.325,350,409	1.7,313,488	0.08056
HMDB0000954	Ferulate	Secondary metabolism	0.136,893,617	3.07,991,372	0.0284	0.035,539,577	0.61,198,299	0.18665

FC, fold change; HMDB, Human Metabolome Database; VIP, Variable Importance in Projection.

a Student’s *t*-test was used for statistical analysis.

Pathway enrichment analysis of these 54 HFD-specific metabolites (Fig. 5G) identified sphingolipid metabolism, glycerophospholipid metabolism, and biosynthesis of unsaturated fatty acids as the top three perturbed pathways. These findings are consistent with our earlier results in Figure 3, where global metabolic reprogramming under HFD also prominently involved lipid remodeling and amino acid dysregulation. These data indicate that early HFD exposure triggers coordinated and reproducible alterations in membrane lipid metabolism and amino acid pathways, which may precede and drive the onset of systemic metabolic dysfunction.

### Serotonin, inosine, spermine, and FIGLU as specific early warning biomarkers for early HFD-induced obesity

**Fig. 6:**
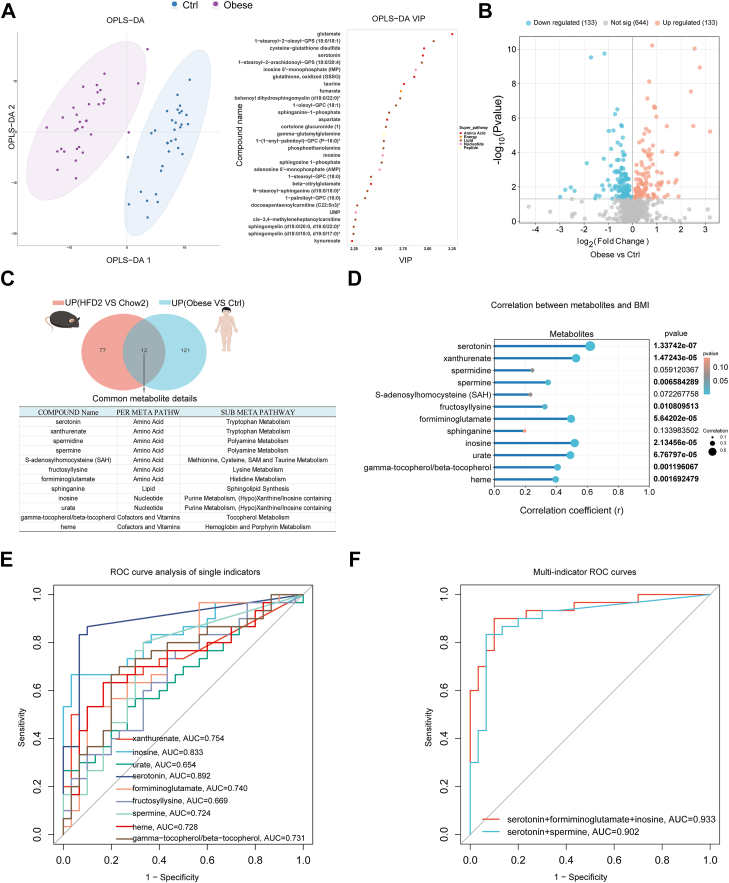
E**arly warning metabolites for obesity.** A: The OPLS-DA plot between obese and control children. B: Volcano map of plasma metabolomics. C: Venn diagram of common upregulated metabolites in week 2 HFD-fed mice (HFD2 vs. Chow2) and obese human subjects. D: Correlation analysis of common metabolites with BMI. E: ROC curves of metabolites. F: ROC curve of combined early biomarker prediction for obesity. OPLS-DA, orthogonal partial least squares-discriminate.

To explore metabolic alterations associated with obesity, we performed untargeted metabolomics on peripheral blood plasma samples collected from 30 obese children and matched controls. The general characteristics of the study participants are summarized in Supplemental Table S1. Orthogonal partial least squares-discriminate analysis revealed a clear separation between the obese and control group, indicating distinct metabolic signatures. The corresponding Variable Importance in Projection plot identified key discriminative metabolites contributing to group separation (Fig. 6A). Volcano plot analysis further revealed that 133 metabolites were significantly downregulated and 133 were significantly upregulated in obese individuals compared with controls (Fig. 6B). Next, we performed a cross-species comparative analysis of differentially upregulated metabolites in HFD-fed mice (HFD2 vs. Chow2) and obese children. Twelve overlapping metabolites were identified (Fig. 6C, Table 2), including serotonin, xanthurenate, spermidine, S-adenosylhomocysteine, sphinganine, inosine, and gamma-tocopherol. We examined the association between these 12 metabolites and BMI in children using linear regression analysis to assess their clinical relevance. Nine metabolites exhibited significant positive correlations with BMI (*P* < 0.05), with serotonin, xanthurenate, inosine, and FIGLU showing the strongest associations (Fig. 6D). Given these metabolites' clinical relevance, we explored the predictive potential of BMI-associated metabolites for detecting obesity. Several metabolites, including serotonin (AUC = 0.892), inosine (AUC = 0.833), FIGLU (AUC = 0.740), and spermine (AUC = 0.724), demonstrated strong discriminatory power (Fig. 6E).

**Table 2:** Common upregulated metabolites in week 2 HFD-fed mice (HFD2 vs. Chow2) and in obese human subjects

HMDB	Metabolites	Sub meta pathway	Mice				Human			
			Average (HFD-2)	Average (chow-2)	FC (HFD/chow)	*P*^a^	Average (Ctrl)	Average (obese)	FC (obese/Ctrl)	*P*^a^
HMDB0000854	FIGLU	Histidine metabolism	82,059,362	25,898,781	3.168	0.003	525,958	716,919	1.363	0.002
HMDB0034879	Fructosyllysine	Lysine metabolism	12,775,619	7,872,055	1.623	0.018	26,914,465	34,152,834	1.269	0.026
HMDB0000939	S-adenosylhomocysteine	Methionine, cysteine, S-adenosylmethionine, and taurine metabolism	31,528	15,017	2.100	0.039	52,562	94,185	1.792	0.026
HMDB0001257	Spermidine	Polyamine metabolism	11,502,780	665,515	17.284	0.014	301,460	334,660	1.110	0.049
HMDB0001256	Spermine	Polyamine metabolism	90,971	15,017	6.058	0.019	77,292	84,731	1.096	0.015
HMDB0000259	Serotonin	Tryptophan metabolism	15,549,651	3,807,971	4.083	0.004	286,531	460,218	1.606	<0.001
HMDB0000881	Xanthurenate	Tryptophan metabolism	538,413	123,266	4.368	<0.001	49,354	76,827	1.557	0.000
HMDB0003178	Heme	Hemoglobin and porphyrin metabolism	14,476,933	5,446,502	2.658	0.027	2,423,575	5,543,359	2.287	0.002
HMDB0001492	Gamma-tocopherol/beta-tocopherol	Tocopherol metabolism	4,021,461	127,637	31.507	0.010	28,938,488	37,678,992	1.302	0.002
HMDB0000269	Sphinganine	Sphingolipid synthesis	5,725,118	1,895,552	3.020	0.057	182,188	235,803	1.294	0.023
HMDB0000195	Inosine	Purine metabolism, (hypo)xanthine/inosine containing	31,647	15,017	2.107	0.039	121,909	1,117,282	9.165	<0.001
HMDB0000289	Urate	Purine metabolism, (hypo)xanthine/inosine containing	163,511,427	43,990,162	3.717	<0.001	176,997,274	201,988,845	1.141	0.026

FC, fold change; HMDB, Human Metabolome Database.

a Student’s *t*-test was used for statistical analysis.

To further enhance diagnostic performance, we evaluated combinations of these metabolites. Among all tested combinations, the panel comprising serotonin, FIGLU, and inosine yielded the highest predictive value, achieving an AUC of 0.933. In addition, metabolites related to tryptophan metabolism (serotonin) and polyamine metabolism (spermine) showed strong predictive potential when combined (AUC = 0.902) (Fig. 6F). Together, these findings identify a combined metabolic signature of serotonin, inosine, FIGLU, and spermine as an early biomarker panel for detecting children at risk of obesity and related metabolic dysfunction.

Furthermore, we analyzed stage-specific metabolic alterations during obesity progression. In mice, 286 metabolites changed exclusively at the late stage of HFD-induced obesity (HFD15), whereas remaining unchanged at the early stage (HFD2). Among them, 67 metabolites overlapped with those altered in obese children. Lipids accounted for 46.27% and amino acids for 32.84% of these metabolites, highlighting amino acid and lipid metabolism as the predominant conserved pathways (Supplemental Fig. S5, Supplemental Table S2).

### Short-term HFD induces transient immune dysregulation

**Fig. 7:**
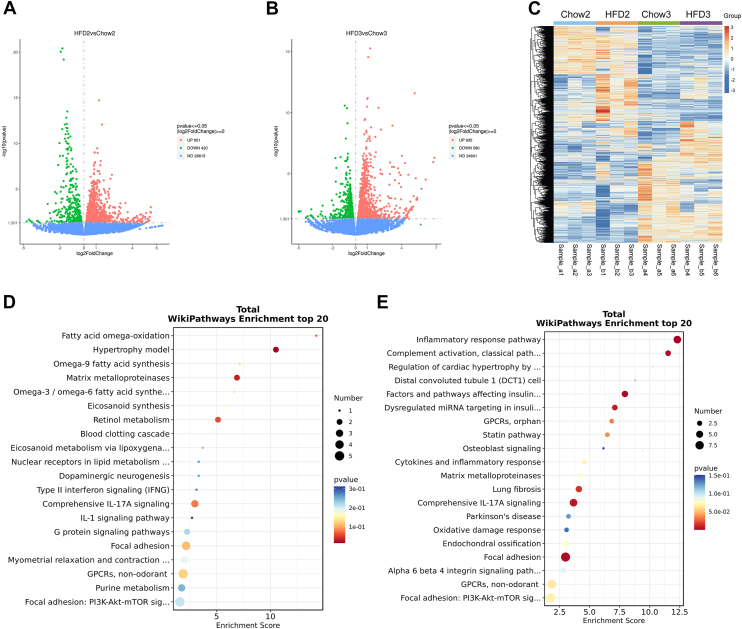
**Transcriptomic profiling of PBMCs under early HFD feeding.** A and B: Volcano plots showing DEGs in PBMCs between HFD-fed and chow-fed mice at 2 weeks (A) and 3 weeks (B). Red and green dots indicate significantly upregulated and downregulated genes, respectively (fold change >2, *P* < 0.05). C: Heatmap displaying gene expression clustering. D and E: WikiPathways enrichment analysis of DEGs between HFD-fed and chow-fed mice at 2 weeks (HFD2 vs. Chow2) (D) and 3 weeks (HFD3 vs. Chow3) (E). DEGs were identified using a fold change >2 and *P* < 0.05. Data are expressed as mean ± SD. ∗*P* < 0.05, ∗∗*P* < 0.01, ∗∗∗*P* < 0.001 versus chow. DEG, differentially expressed gene; NS, not significant.

Recent studies have shown that switching from a fiber-rich habitual diet to a high-energy “feast” disrupts immune homeostasis (14, 15, 16). To explore early immunologic alterations induced by HFD, we performed RNA-Seq on PBMCs from mice after 2 and 3 weeks of HFD feeding. Volcano plots revealed numerous differentially expressed genes between HFD and chow groups at both time points (Fig. 7A and B). Hierarchical clustering further distinguished HFD-fed mice from controls, indicating early transcriptional remodeling (Fig. 7C). Pathway enrichment analysis using WikiPathways showed distinct time-dependent changes. At week 2, HFD primarily enriched metabolic and lipid-related pathways, including fatty acid ω-oxidation, along with early activation of interleukin 17A (IL-17A) and type II interferon signaling (Fig. 7D). By week 3, enrichment shifted toward inflammation-related pathways, such as complement activation, inflammatory response, oxidative stress, and IL-17A signaling (Fig. 7E), marking a transition from metabolic stress to systemic inflammation.

## Discussion

These findings indicate that significant glucose metabolic dysregulation occurs before reaching the diagnostic threshold for obesity. Consistent with our results, Kenichi Sakamoto *et al.* (5) found that mice fed with HFD for 10 days showed elevated blood glucose in both fasting and eating states and impaired glucose tolerance. We speculate that this glucose metabolism disorder may be driven by cellular stress directly caused by HFD (such as lipid and amino acid metabolism inflammatory responses) rather than secondary to fat accumulation. Metabolomic profiling after 2 weeks of HFD feeding revealed marked alterations in fatty acid and amino acid metabolism, suggesting that metabolic reprogramming precedes glucose intolerance. Analysis of the metabolic signature at 2 weeks of HFD feeding demonstrated reduced acylcarnitine levels, indicating potential impairments in mitochondrial fatty acid transport and β-oxidation (17). Concurrently, glycerophospholipid metabolism exhibited marked changes: phosphatidylcholine and phosphatidylinositol were consistently downregulated, whereas phosphatidylethanolamine, lysophospholipids, and sphingosines were upregulated, shifts consistent with a transition toward proinflammatory lipids (18, 19). In addition, despite ceramides being established mediators of insulin resistance and inflammation (20, 21), ceramide metabolites transiently decreased at week 2. This paradoxical reduction may arise from compensatory sphingolipid adaptations to mitigate cellular stress, potentially as a protective mechanism against apoptosis or excessive inflammation.

Another key finding of this study is that remodeling and sustained activation of the tryptophan-polyamine metabolic pathways represent hallmarks of early metabolic reprogramming induced by HFD exposure. Short-term HFD feeding triggers tissue-specific adaptations, with adipose tissues—particularly eWAT and BAT—serving as the main sites of early reprogramming in these pathways. Tryptophan and polyamine metabolic pathways converge at aryl hydrocarbon receptor a (AhR-a), a ligand-activated transcription factor widely expressed in immune and metabolic tissues (22, 23). Critically, tryptophan metabolites are known to activate the AhR, modulating the Th17/regulatory T-cell balance (24). The shunting of tryptophan toward kynurenine is mediated by IDO1, and kynurenine can interact with AhR to promote the increase of regulatory T cell and inhibit Th17 differentiation (25, 26). Meanwhile, polyamines act both as metabolic intermediates and immunoregulatory signals. Polyamine metabolites promote β-adrenergic receptor-dependent release of FFAs from adipocytes, and excessive lipolysis drives ectopic lipid deposition, oxidative stress, and insulin resistance (27). In our study, upregulated polyamine metabolism during early HFD stages coincided with increased FFA flux. Polyamine biosynthesis is regulated by ornithine decarboxylase, a direct transcriptional target of AhR (28). Integrating these findings, our transcriptomic analysis revealed significant enrichment of the IL-17A signaling pathway in PBMCs, implying that AhR-mediated immunometabolic crosstalk constitutes a central regulatory axis connecting HFD exposure to early glucose intolerance.

Taken together, these findings position the tryptophan/polyamine-AhR axis as a critical regulatory node during the initial phases of HFD-induced metabolic disruption. This pathway highlights mechanistic links between dietary stress and immune-metabolic dysfunction and provides a potential foundation for developing early biomarkers and therapeutic strategies to combat childhood obesity.

Serotonin, inosine, and FIGLU were identified as sensitive dysregulated metabolites in both HFD-fed mice and obese children. Serotonin (5-hydroxytryptamine [5-HT]) is a key neurotransmitter and hormone synthesized from the essential amino acid tryptophan by the rate-limiting enzyme tryptophan hydroxylase. Increasing evidence implicates disrupted tryptophan-5-HT metabolism in gut microbiota-host interactions that contribute to metabolic disorders, such as obesity and diabetes. Gut-derived 5-HT, regulated by the intestinal microbiota, has been positively associated with metabolic syndrome, as circulating 5-HT levels are elevated in obese individuals (29). Conversely, pharmacological inhibition or genetic deletion of gut-derived 5-HT synthesis protects HFD-fed mice from obesity and glucose intolerance (30). Consistent with these findings, our study demonstrated early elevations of peripheral 5-HT in HFD-fed juvenile mice, which correlated positively with BMI in obese children. Inosine, a purine nucleoside produced by adenosine deamination, functions as a multifunctional metabolic and immunomodulatory molecule. Mechanistically, inosine activates cAMP signaling to promote adipose thermogenesis and induces uncoupling protein 1 expression in brown and beige adipocytes (31). It also enhances energy expenditure and alleviates obesity-associated inflammation through macrophage A2A receptor signaling (32). In our study, circulating inosine levels were elevated early in HFD-fed mice and positively correlated with BMI in obese children. This early increase may reflect a compensatory response to mitochondrial stress or inflammation that fails to offset the metabolic burden of overnutrition. FIGLU is a sensitive marker of folate metabolism derived from the histidine degradation pathway (33). Notably, increased FIGLU levels were among the top predictors of childhood obesity in our data. Given folate's established links to immune development (34), early HFD-induced FIGLU accumulation may be a harbinger of impaired metabolic plasticity and immune programming during critical developmental windows. The traditional screening strategy based on BMI and fasting blood glucose may miss the optimal intervention period. Remarkably, when analyzed as combined biomarkers, serotonin, inosine, and FIGLU demonstrated superior diagnostic performance, significantly better than any single metabolite.

## Conclusions

Overall, the onset of hyperglycemia represents a critical early window in obesity progression, whereas metabolic reprogramming occurs even earlier and may serve as an initiating event. Integrating plasma metabolomics data from obese children, serotonin, inosine, FIGLU, and spermine were proposed as potential early biomarkers. However, several limitations should be noted. First, female animals were not included, preventing evaluation of sex-specific metabolic responses. Second, this study primarily relied on multiomics analyses. Therefore, this study aims to call for more research to focus on the metabolic impact of an HFD on children in their early years.

## Ethics Approval and Consent to Participate

The study was approved by the Ethics Committee of the Children's Hospital, Zhejiang University School of Medicine (approval no.: 2021-IRB-301). All animal protocols are followed by the Animal Ethics Committee of Tongren Hospital, China (reference number: A2023-079-01, dated: August 16, 2024). The studies in this work abide by the Declaration of Helsinki principles.

## Data availability

The data used to support the findings of this study are available from the corresponding author upon request.

## Supplemental data

This article contains supplemental data.

## Conflict of interest

The authors declare that they have no conflicts of interest with the contents of this article.

## Figures and Tables

**Supplemental Figure S5:**