# Supplementary material for: Tryptophan and polyamine metabolism dysregulation serves as an early marker of high-fat diet-induced glucose intolerance
Source: J Lipid Res. 2026 Jan 13;67(2):100980. doi: 10.1016/j.jlr.2026.100980 (PMC12907098; doi:10.1016/j.jlr.2026.100980)
Supplement: Supplemental Table S1 [file mmc1.docx]

Supplementary table 1. Baseline characteristics of controls and obese participants

| **Group^a^** | **Normal weight (n = 30)** | **Overweight/Obesity**  **(n = 30)** | ***P*-value^b^** |
| --- | --- | --- | --- |
| **Sex** |  |  | 0.284 |
| Boys | 17 (56.7 %) | 21 (70.0 %) |  |
| Girls | 13 (43.3 %) | 9 (30.0 %) |  |
| Age (Years) | 11.47±0.32 | 10.20±0.35 | 0.01 |
| Height (cm) | 150.20±2.50 | 144.95±2.23 | 0.123 |
| Weights (kg) | 37.80±1.80 | 53.99±2.56 | <0.001 |
| BMI (kg/cm^2^) | 16.46±0.37 | 25.29±0.61 | <0.001 |

^a^Represented as “median (SEM)” or “number of samples (percentage)”.

^b^Compared between controls and obese participants categorical variables (*χ*2-test) and continuous variable (Mann-Whitney U test).
