# Supplementary material for: Tryptophan and polyamine metabolism dysregulation serves as an early marker of high-fat diet-induced glucose intolerance
Source: J Lipid Res. 2026 Jan 13;67(2):100980. doi: 10.1016/j.jlr.2026.100980 (PMC12907098; doi:10.1016/j.jlr.2026.100980)
Supplement: Supplemental Information [file mmc3.docx]

**Supplementary Information**

**Tables**

**Supplementary information, Table S1. Baseline characteristics of controls and obese participants**

| **Group^a^** | **Normal weight (n = 30)** | **Overweight/Obesity**  **(n = 30)** | ***P*-value^b^** |
| --- | --- | --- | --- |
| **Sex** |  |  | 0.284 |
| Boys | 17 (56.7 %) | 21 (70.0 %) |  |
| Girls | 13 (43.3 %) | 9 (30.0 %) |  |
| Age (Years) | 11.47±0.32 | 10.20±0.35 | 0.01 |
| Height (cm) | 150.20±2.50 | 144.95±2.23 | 0.123 |
| Weights (kg) | 37.80±1.80 | 53.99±2.56 | <0.001 |
| BMI (kg/cm^2^) | 16.46±0.37 | 25.29±0.61 | <0.001 |

^a^Represented as “median (SEM)” or “number of samples (percentage)”.

^b^Compared between controls and obese participants categorical variables (*χ*2-test) and continuous variable (Mann-Whitney U test).

**Supplementary information, Table S2. Metabolites specifically altered in the late stage of obesity**

**Figures**





**Supplementary information, Figure S1. Body weight and fat mass increase during HFD feeding**

(A) Body weight. 4-week-old male C57BL/6J mice were fed an HFD (n = 10) or chow diet (n = 10) for 15 weeks. (B) Food intake. (C) Representative images of DIO model mice at the endpoint. (D) Effects of HFD on BAT weight in mice. Each symbol represents an individual mouse. Data are shown as mean ± SEM. **P* < 0.05, ***P* < 0.01, ****P* < 0.001 vs. chow, NS, not significant. HFD, high-fat diet; eWAT, epididymal adipose tissue; iWAT, inguinal adipose tissue; BAT, brown adipose tissue.





**Supplementary information, Figure S2. Long-term HFD feeding induced abnormal glucose metabolism and insulin resistance.**

(A–H) Effects of HFD on glucose intolerance in vivo. Male C57BL/6J mice (n = 10 per group) were fed an HFD or chow for 6 weeks, 8 weeks, 10 weeks, or 15 weeks. After 12 h (GTT, ITT) fasting at the endpoint, glucose levels and AUC were determined using the GTT and ITT. Data are expressed as mean ± SD. **P* < 0.05, ***P* < 0.01, ****P* < 0.001 vs. chow, NS, not significant. HFD, high-fat diet; eWAT, epididymal adipose tissue; iWAT, inguinal adipose tissue; BAT, brown adipose tissue; GTT, Glucose tolerance test; ITT, insulin tolerance test; AUC, area under the curve.



**Supplementary information, Figure S3. Plasma metabolic alterations after 15 weeks of HFD**

(A) Volcano plots showing significantly altered metabolites in mice fed a chow or HFD for 15 weeks. (B) Biological classification of altered metabolites is categorized by superclasses such as lipids, amino acids, carbohydrates, and cofactors. (C) Relative class distribution of significantly altered lipid-related metabolites. (D) Relative class distribution of significantly altered amino acid-related metabolites. HFD, high-fat diet.


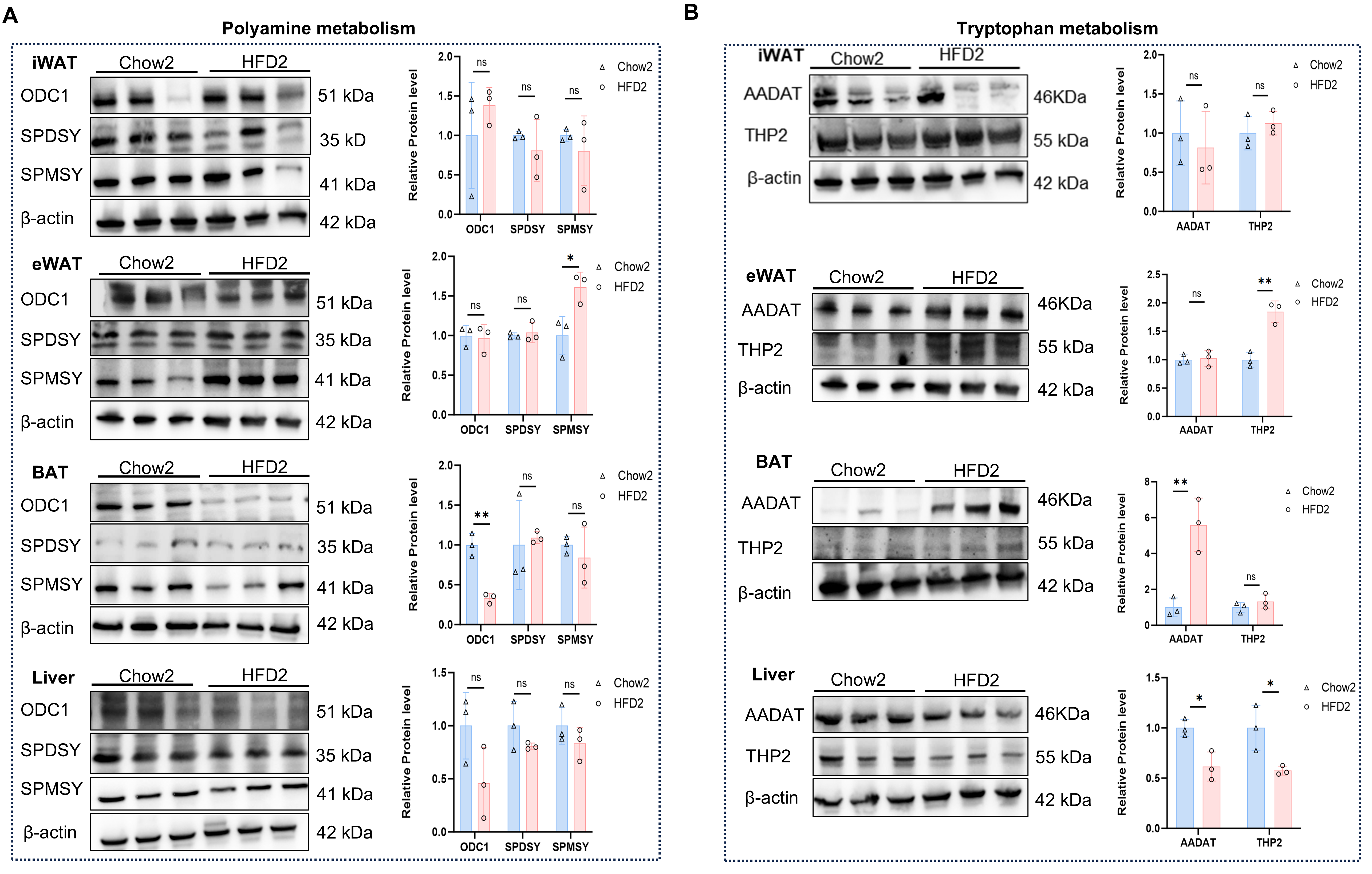


**Supplementary information, Figure S4. Polyamine and tryptophan metabolism enzyme expression after 2-week HFD**

A: Key enzymes involved in polyamine metabolism (ODC1, SPDSY, and SPMSY) in iWAT, eWAT, BAT, and liver; B: Tryptophan metabolism (AADAT and THP2) in iWAT, eWAT, BAT, and liver. iWAT, inguinal white adipose tissue; eWAT, epididymal white adipose tissue; BAT, brown adipose tissue; ODC1, ornithine decarboxylase 1; SPDSY, spermidine synthase; SPMSY, spermine synthase; AADAT, aminoadipate aminotransferase; TPH2, tryptophan hydroxylase 2. *P* < 0.05, **P* < 0.05, ***P* < 0.01; ns, not significant.


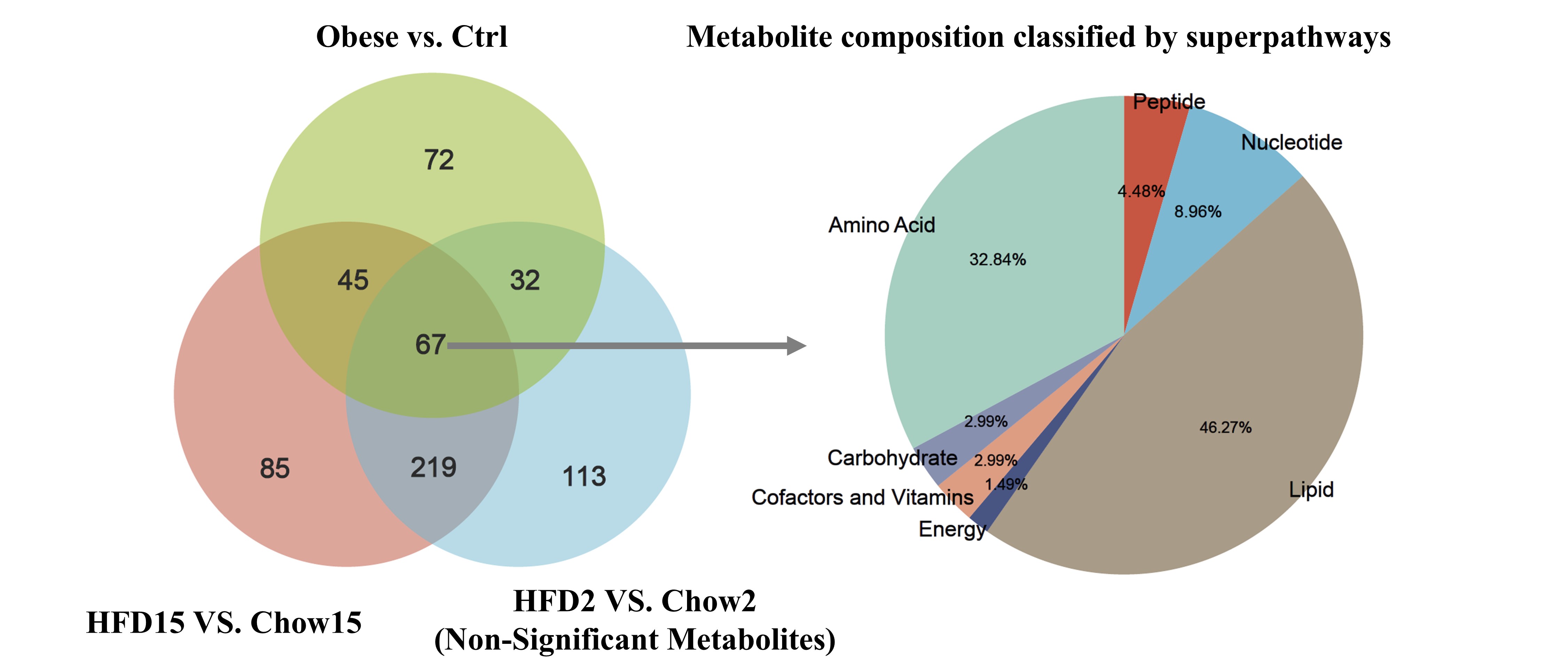


**Supplementary information, Figure S5. Identification of obesity-specific metabolites by cross-stage and cross-species comparison**
